# Basolateral BMP Signaling in Polarized Epithelial Cells

**DOI:** 10.1371/journal.pone.0062659

**Published:** 2013-05-13

**Authors:** Masao Saitoh, Takuya Shirakihara, Akira Fukasawa, Kana Horiguchi, Kei Sakamoto, Hiroshi Sugiya, Hideyuki Beppu, Yasuyuki Fujita, Ikuo Morita, Kohei Miyazono, Keiji Miyazawa

**Affiliations:** 1 Department of Biochemistry, Interdisciplinary Graduate School of Medicine and Engineering, University of Yamanashi, Chuo, Yamanashi, Japan; 2 Division of Metastasis & Invasion Signaling, National Cancer Center Research Institute, Chuo-ku, Tokyo, Japan; 3 Department of Molecular Pathology, Graduate School of Medicine, The University of Tokyo, Bunkyo-ku, Tokyo, Japan; 4 Section of Oral Pathology, International Research Center for Molecular Science in Tooth and Bone Diseases, Graduate School of Medical and Dental Sciences, Tokyo Medical and Dental University, Bunkyo-ku, Tokyo, Japan; 5 Laboratory of Veterinary Biochemistry, Nihon University College of Bioresource Sciences, Fujisawa, Kanagawa, Japan; 6 Department of Clinical Laboratory and Molecular Pathology, Graduate School of Medicine and Pharmaceutical Science for Research, University of Toyama, Toyama, Japan; 7 Division of Molecular Oncology, Institute for Genetic Medicine, Hokkaido University, Sapporo, Hokkaido, Japan; 8 Department of Cellular Physiological Chemistry and Global Center of Excellence (GCOE) Program, International Research Center for Molecular Science in Tooth and Bone Diseases, Graduate School of Medical and Dental Sciences, Tokyo Medical and Dental University, Bunkyo-ku, Tokyo, Japan; Institut Curie, France

## Abstract

Bone morphogenetic proteins (BMPs) regulate various biological processes, mostly mediated by cells of mesenchymal origin. However, the roles of BMPs in epithelial cells are poorly understood. Here, we demonstrate that, in polarized epithelial cells, BMP signals are transmitted from BMP receptor complexes exclusively localized at the basolateral surface of the cell membrane. In addition, basolateral stimulation with BMP increased expression of components of tight junctions and enhanced the transepithelial resistance (TER), counteracting reduction of TER by treatment with TGF-β or an anti-tumor drug. We conclude that BMPs maintain epithelial polarity via intracellular signaling from basolaterally localized BMP receptors.

## Introduction

Tubular epithelial tissues, including the small intestine, kidneys, and exocrine glands, as well as circulatory tissues such as blood vessels, are lined by epithelia consisting of polarized epithelial cells. The polarization of epithelial cells is essential for separating the lumens of these tissues from the stroma, and for orienting the vectorial transport of ions and fluids containing various growth factors and cytokines. Breakdown of epithelial polarity by chronic inflammation or injuries results in compromised barrier function, leading to mucosal damage, as in the cases of Crohn’s disease and renal fibrosis, and eventually in tumorigenesis by epithelial cells near sites of damage in response to allopatric molecules passed from the lumen [1], [2]. Thus, the signals involved in maintaining epithelial polarity play crucial roles in recovery from damage to epithelial cells and protection from epithelial-related diseases.

The plasma membrane of epithelial cells is physically separated by tight junctions into two surfaces, apical and basolateral, with distinct lipid and protein compositions. These two surfaces play important roles in determining the polarity and function of epithelial cells [3], [4]. Polarized targeting to the basolateral surface is largely dependent on interactions between the sorting motifs of basolateral cargo proteins with the µ subunit of clathrin adaptor protein (AP) complexes. There are four types of AP complexes; among them, the AP1 and AP4 complexes are involved in basolateral sorting through binding to distinct types of cytosolic motif known to mediate basolateral transport; however, the detailed role of AP4 remains poorly understood [5], [6]. The µ1A subunit, which is expressed in all cell types, assembles to generate the AP1A complex; the µ1B subunit, expressed only in epithelial cells, is a component of the AP1B complex, which shares the other subunits of AP1A. Recent studies using gene-targeted mice and *in vitro* experiments have revealed that AP1A as well as AP1B are capable of basolateral delivery of cargo proteins [7]–[9].

Cytokines of the transforming growth factor (TGF)-β family, such as TGF-βs and bone morphogenetic proteins (BMPs), are critical for embryonic development and a large number of other biological processes. BMPs and TGF-βs have been considered as key regulators of bone metabolism and epithelial-mesenchymal transition (EMT) that occurs during organogenesis and in cancer progression and fibrosis [10]–[12]. BMPs also regulate cancer progression by promoting EMT and angiogenesis in certain kinds of cancer cells [13], [14]. However, BMPs reverse TGF-β–induced EMT in renal tubular and mammary ductal epithelial cells during chronic injuries. Furthermore, systemic administration of BMPs leads to repair of severely damaged renal tubular epithelial cells in association with reversal of chronic renal injury [15].

To date, numerous previous *in vitro* and *in vivo* studies have demonstrated a range of biological effects of BMPs in a variety of mesenchymal cells. Recently, the pathophysiological importance of BMPs in epithelial cells has been revealed [16], [17], but their roles in polarized epithelial cells are less well understood. In this study, we investigated BMP signaling in polarized epithelial cells. We observed that BMPs added to the basolateral side, where the receptor BMPR-II is exclusively localized, induced phosphorylation of Smad1/5/8 and activated transcription of their target genes. By contrast, TGF-β added to the apical side induced phosphorylation of Smad2. Furthermore, BMPR-II interacts with AP1 µ1, and AP1 µ1A siRNA in AP1 µ1B-defective cells resulted in disordered BMP signaling input from the apical surface. BMP increased transepithelial resistance (TER) and expression of claudin proteins, and inhibited the EMT phenotype induced by TGF-β. These findings suggest that BMP maintains epithelial polarity via signaling from BMP receptors localized at the basolateral surface.

## Materials and Methods

### Cell Culture

Madin-Darby canine kidney polarized epithelial (MDCK-I and MDCK-II) cells, non-polarized normal mammary epithelial (NMuMG) cells, and mouse muscle myoblast (C2C12) cells were obtained from the American Type Culture Collection (ATCC; Manassas, VA), and cultured in Dulbecco’s modified Eagle’s medium (DMEM) supplemented with 4.5 g/l glucose, 10% fetal bovine serum (FBS), 100 U/ml penicillin, and 100 µg/ml streptomycin. Insulin (10 µg/ml) was added to the culture media of NMuMG cells. All cells were grown in a 5% CO_2_ atmosphere at 37°C. Porcine kidney polarized epithelial (LLC-PK1) cells were kindly provided by Dr. H. Ohno (Riken Institute) [18], [19]. MDCK-Ras cells stably expressing GFP-tagged Ras under the control of a tetracycline-inducible (Tet-ON) promoter (denoted MDCK-Ras cells) were described previously [20]. MDCK cells expressing Tet-Off transactivator protein were obtained from Clontech Laboratories (Berkley, CA) and transfected with the pBABE plasmid, which encodes HA-tagged BMPR-II under the control of a Tet-repressible (Tet-OFF) promoter (denoted MDCK-BR2 cells).

### Reagents and Antibodies

Recombinant human TGF-β1, BMPs, and HGF were purchased from R&D Systems (Minneapolis, MN). The following antibodies were used: mouse monoclonal anti–α-tubulin (Sigma-Aldrich, Saint Louis, MO); anti-Smad2/3, -BMPR2, -EGFR, –E-cadherin, and –ZO-1 (BD Transduction Laboratory, Franklin Lakes, NJ); anti–claudin-1 and –claudin-4 (Invitrogen, Carlsbad, CA); anti-AP2 µ (Novus Biologicals, Littleton, CO); rabbit polyclonal anti–phospho-Smad2 (Millipore, Bedford, MA); anti–Smad1, anti–phospho-Smad1/5/8, and anti–phospho-Erk1/2 (Cell Signaling, Beverly, MA); anti-AP1 µ1 (Proteintech Group, Chicago, IL); and rat monoclonal anti-HA (3F10) (Roche, Indianapolis, IN). Rhodamine-conjugated phalloidin and TOPRO were from Molecular Probes (Eugene, OR). Monoclonal anti-HA covalently linked to agarose was from Sigma-Aldrich.

### Immunoprecipitation, Immunoblotting, and Luciferase Assay

The procedures used for immunoprecipitation, immunoblotting, and luciferase assay were previously described [21]. Immunodetection was performed with the ECL blotting system (Amersham Bioscience, Piscataway, NJ) and Luminescent Image Analyzer (LAS4000, Fujifilm, Tokyo, Japan). α-tubulin and Smad1 were used as loading controls. For luciferase assays, cells were seeded into 60-mm plates and transfected with a TGF-β–responsive reporter, (CAGA)_12_-MLP-Luc, or a BMP–responsive reporter, BRE-Luc. Twelve hours later, cells were trypsinized and re-seeded in duplicate into 12-well Transwell plates (0.4-µm pore size; BD Falcon, Franklin Lakes, NJ). After the cells were grown to confluence, TGF-β (1 ng/ml) or BMP-4 (20 ng/ml) was added to the apical or basolateral side, and the cells were incubated for 2 h. Luciferase activities were determined using the Dual-Luciferase Reporter Assay system (Promega, San Luis Obispo, CA) using a SpectraMax luminometer (Molecular Devices, Sunnyvale, CA). Luciferase activity was normalized to sea pansy luciferase activity expressed from the cotransfected plasmid phRL-TK (Promega).

### RNA Interference

Short interfering RNAs (siRNAs) were transfected into cells according to the protocol recommended for the HiPerFect reagent (Qiagen, Valencia, CA). LLC-PK1 cells were transiently transfected with siRNAs against pig Ap1 µ1A: #1, UACUCCUGCAGGAUCUUGCUGUCUG; #2, ACUCGAUGACGUCCAAGAACACCUC (Stealth RNAi; Invitrogen). The final concentration of each siRNA was 10 nM. LLC-PK1 cells were transiently transfected with either control siRNA (Stealth RNAi 12935-300; Invitrogen) or AP1 µ1A siRNA in 60-mm plates. After 12 h, the cells were trypsinized, seeded on Transwell plates, and grown to confluence. BMP4 (20 ng/ml) was added to apical or basolateral media, and the cells were incubated for 45 min. The cells were harvested and assayed for immunoblot analyses using the indicated antibodies.

### RNA Extraction and RT-PCR

Total RNA was extracted and analyzed by quantitative or conventional RT-PCR analyses as previously described [21]. The specificity of the detected signals was confirmed by a dissociation curve, which consisted of a single peak. Values were normalized to those of glyceraldehyde 3-phosphate dehydrogenase (GAPDH). The RT-PCR primer sequences used are shown in Table S1.

### 3D Culture in Matrigel

Matrigel (BD Biosciences, San Jose, CA) was added onto cover glasses and allowed to set at 37°C for 30 min. MDCK-BR2 cells (5.0×10^4^ cells per 24-well plate) pre-cultured in the presence or absence of tetracycline for 48 h were suspended in 2.0% Matrigel in complete medium and layered on top of the gel. Three days later, the gels containing MDCK cells were fixed with 4% paraformaldehyde in PBS. After staining with anti-HA antibody, rhodamine-phalloidin, and TOPRO, fluorescence was examined using a confocal laser scanning microscope (Olympus, Tokyo, Japan).

### Transepithelial Resistance (TER) and Cell Counting

MDCK cells were seeded to form monolayers in duplicate Transwells (0.4-µm pore size) in 12-well plates in complete media containing 3% FBS in the presence or absence of BMP-4. After TER was measured using the MILLICELL-ERS (Millipore, Billerica, MA), cells were trypsinized and counted using a hemocytometer.

### Biotinylation

MDCK-I cells were grown to confluence on Transwells (0.4-µm pore size) in 6-well plates. After cell-surface biotinylation was performed from either apical or basolateral sides, using the Pierce Cell Surface Protein Isolation Kit (Thermo Scientific, Rockford, IL), the cells were scraped with a rubber policeman in buffer (1% Nonidet P-40, 20 mM Tris-HCl (pH 7.4), 150 mM NaCl, 5 mM EDTA, 1 mM EGTA, and protease inhibitors (Nacalai Tesque, Kyoto, Japan), and centrifuged at 15000 rpm for 20 min at 4°C. Equal amounts of protein were subjected to SDS-PAGE or incubated with Streptavidin Sepharose 4B (GE Healthcare Biosciences, Piscataway, NJ) for 1 h to isolate biotinylated proteins. The avidin-bound complexes were washed with the same buffer and subjected to SDS gel electrophoresis.

## Results and Discussion

### BMP Signaling from the Basolateral Side in Polarized Epithelial Cells

**Figure 1:**
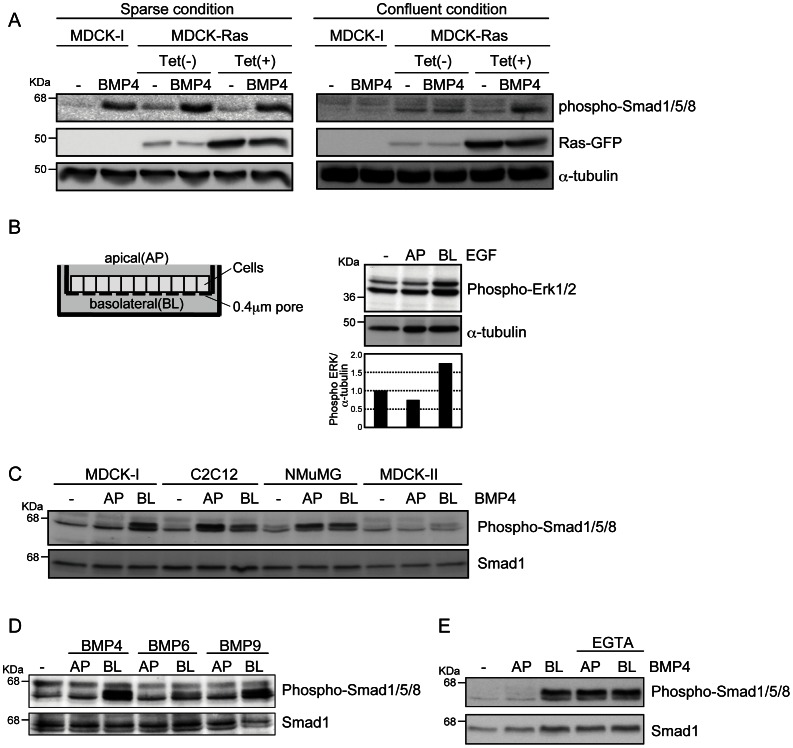
BMP signaling from the basolateral side in polarized epithelial cells. (**A**) Phosphorylation of Smad1/5/8 induced by BMP4 was examined by immunoblot analyses. MDCK-I and MDCK-Ras cells were seeded at the density of 2.0×10^5^ (sparse) or 1.0×10^6^ (confluent) cells in 6-well plates. Twenty four hours later, the cells were treated with 20 ng/ml BMP4 for 45 min. (**B**) MDCK-I cells were grown to confluence on Transwell plates (left) and treated with 10 ng/ml of EGF from the apical (AP) or basolateral (BL) sides for 15 min. Phosphorylation of Erk1/2 induced by EGF was examined by immunoblot analyses. The ratio of phospho-Erk1/2 to α-tubulin was validated by densitometric analysis and shown at the bottom. (**C, D,** and **E**) MDCK-I, C2C12, NMuMG, and MDCK-II cells were grown to confluence on Transwell plates and treated with BMPs from the apical (AP) or basolateral (BL) sides for 45 min. Phosphorylation of Smad1/5/8 induced by BMP4 (20 ng/ml), BMP6 (20 ng/ml), and BMP9 (2 ng/ml) was examined by immunoblot analyses. EGTA (1 mM) was added 3 h before treatment with BMP4 (E).

To investigate BMP signaling in polarized epithelial cells, we treated MDCK-I cells with BMP4 under sparse or confluent conditions in monolayer culture. In the sparse condition, BMP4 induced phosphorylation of Smad1/5/8 (i.e., the BMP receptor–regulated Smads) in MDCK-I cells, whereas it did not have this effect in the confluent condition (Fig. 1A). Next, we investigated MDCK cells stably transfected with GFP-tagged Ras under the control of a tetracycline (Tet)-inducible promoter (MDCK-Ras cells). MDCK-Ras cells in Tet-free media exhibited typical epithelial morphologies, including a cobblestone-like appearance, whereas induction of Ras protein by addition of Tet led cells to adopt an elongated morphology and impaired cell–cell contact through the disorganization of adherens- and tight-junction proteins (Fig. S1) [20]. In sparse conditions with or without Tet, MDCK-Ras cells revealed phosphorylation of Smad1/5/8 in response to BMP4 (Fig. 1A). At confluence, phosphorylation of Smad1/5/8 by BMP4 was observed only in cells expressing Ras (Fig. 1A). These findings suggest that BMP signaling input in polarized epithelial MDCK cells is highly dependent on cell-culture conditions.

To investigate this possibility, MDCK-I cells were seeded onto Transwell plates and grown to confluence to form polarized monolayers (Fig. 1B, left). Full polarization of MDCK-I cells used in this study was confirmed by stimulation with epidermal growth factor (EGF) at the apical or basolateral side (Fig. 1B, right), because EGF receptor (EGFR) is well known to be expressed only at the basolateral surface [22]. As with EGF, BMP4 was administrated from either the apical or basolateral side, and phosphorylation of Smad1/5/8 was assessed by immunoblot analyses. Treatment with BMP4 from the basolateral side induced phosphorylation of Smad1/5/8 in MDCK-I cells, whereas treatment from the apical side did not (Fig. 1C). MDCK-II cells, another MDCK subline with typical polarized epithelial morphology, exhibited a similar profile of Smad1/5/8 phosphorylation, albeit to a lesser extent. On the other hand, non-polarized epithelial NMuMG cells and myoblast C2C12 cells exhibited Smad1/5/8 phosphorylation in response to addition of BMP4 from either side (Fig. 1C). In addition to BMP4, treatment with BMP6 and BMP9 from the basolateral side also resulted in phosphorylation of Smad1/5/8 in MDCK-I cells (Fig. 1D). Treatment with EGTA impaired cell–cell contact through dissociation of adhesion of adherens and tight junctions, as reported [23], and resulted in Smad1/5/8 phosphorylation in response to BMP4 added from the both apical and basolateral sides (Fig. 1E). These findings indicate that BMPs selectively transmit their signals only from the basolateral surface in polarized epithelial cells.

### Basolateral Expression of BMPR-II in Polarized Epithelial Cells

**Figure 2:**
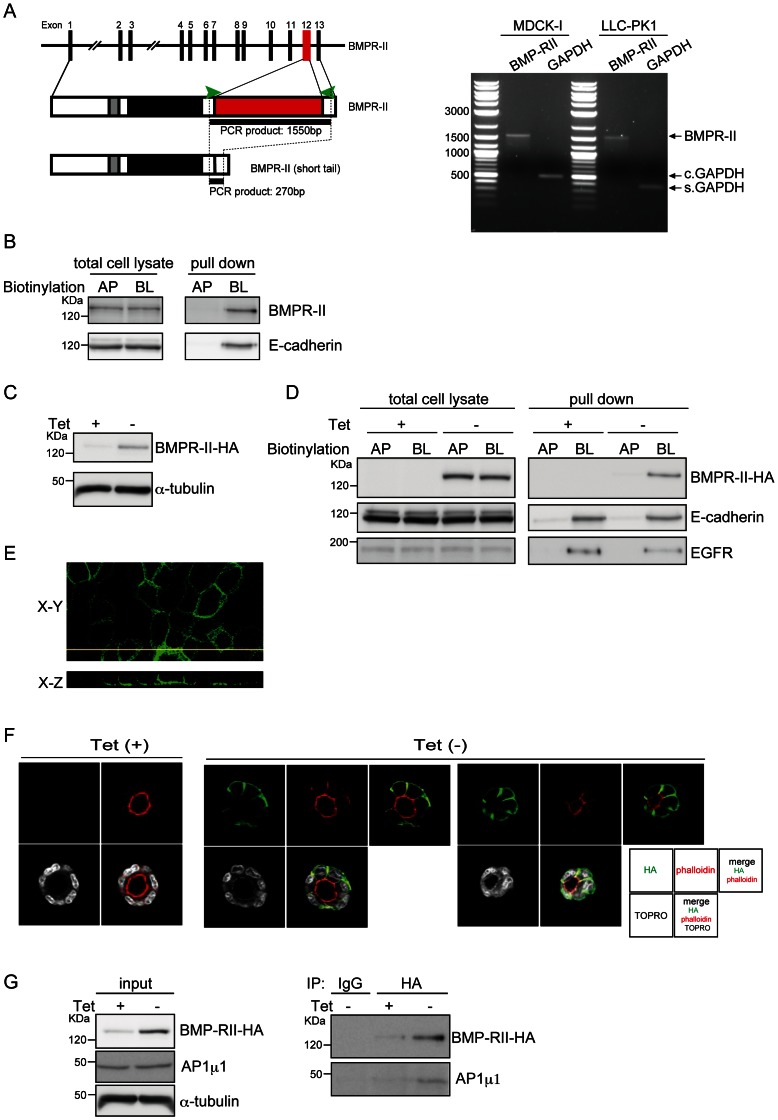
Basolateral localization of BMPR-II. (**A**) A schematic illustration of the BMPR-II gene and protein, containing the transmembrane (grey) and kinase (black) domains and the region encoded by alternative exon 12 (red), is shown on the left. The primers used for PCR are shown as green arrowheads. Expression of alternative-splicing variants of BMPR-II in MDCK-I and LLC-PK1 cells was examined by RT-PCR. GAPDH was used as internal control for *Canis lupus familiaris* MDCK-I cells (c. GAPDH) and *Sus scrofa* LLC-PK1 cells (s. GAPDH). (**B**) After MDCK-I cells cultured on Transwells in a confluent condition were biotinylated from apical (AP) or basolateral (BL) sides, equal amounts of total proteins from each surface were subjected to SDS-PAGE (total cell lysate), or incubated with Streptavidin Sepharose 4B to isolate biotinylated proteins (pulldown). E-cadherin, a representative basolateral protein, was used as a control. (**C**) After withdrawal of tetracycline (Tet) from the culture media, MDCK-BR2 cells were lysed and subjected to immunoblot analysis with an antibody against HA. (**D**) MDCK-BR2 cells were seeded on Transwell plates to reach confluence in the presence or absence of tetracycline (Tet), and biotinylated from the apical (AP) or basolateral (BL) sides. Equal quantities of proteins were subjected to SDS-PAGE (total cell lysate), or incubated with Streptavidin Sepharose 4B to isolate biotinylated proteins, followed by SDS-PAGE (pulldown). E-cadherin and EGFR were used as representative basolateral proteins. (**E**) MDCK-BR2 cells were stained with an anti-HA antibody (green). XY (horizontal) and XZ (vertical) sections are shown in the top and bottom panels, respectively. (**F**) MDCK-BR2 cells cultured in Matrigel in the presence or absence of tetracycline (Tet) were stained with an anti-HA antibody (green), rhodamine-phalloidin (red), and TOPRO (white), followed by fluorescence imaging using a confocal laser scanning microscope. Micrographs of the two independent colonies were taken in the absence of Tet. (**G**) After MDCK-BR2 cells were cultured in the presence or absence of tetracycline (Tet), cell lysates were assayed for protein concentration and then subjected to immunoprecipitation. Co-purified AP1 µ1 was detected by immunoblotting with an anti-AP1 µ1 antibody. The expression levels of BMPR-II-HA, AP1 µ1, and α-tubulin in the same lysates were verified by immunoblotting.

BMPs transmit their signals via heteromeric complexes of two distinct receptors, type-I and type-II. BMPR-II mRNA has two alternative splicing variants in human and mouse: the longer form contains a long carboxy-terminal tail, whereas the shorter form lacks exon 12 encoding most of the tail region (Fig. 2A, left) [24]. RT-PCR analysis revealed that only the longer form of BMPR-II can be detected in MDCK-I and LLC-PK1, a porcine kidney–derived polarized epithelial cell line (Fig. 2A, right). Cell-surface biotinylation analysis revealed that, as with E-cadherin, biotinylated BMPR-II could be detected in MDCK-I cells by labeling from the basolateral side (Fig. 2B), indicating that endogenous BMPR-II is localized basolaterally in polarized epithelial cells.

To examine the molecular mechanisms of basolateral sorting of BMPR-II, we established MDCK cells stably transfected with an HA-tagged allele of the longer form of BMPR-II (BMPR-II-HA) under the control of a Tet-repressive promoter (MDCK-BR2 cells), because the quality of commercially available anti–BMPR-II antibodies was not suitable for immunoprecipitation and immunohistochemical analyses. After withdrawal of Tet from culture media, the level of BMPR-II-HA was clearly upregulated (Fig. 2C). Similar to endogenous E-cadherin and EGFR, overexpressed BMPR-II-HA was properly distributed to the basolateral surface, as determined by cell-surface biotinylation analysis (Fig. 2D), immunohistochemical analyses using perpendicular sections (Fig. 2E), and cultivation in Matrigel in a 3D-culture model (Fig. 2F). Thus, as is the case for endogenous proteins, overexpressed BMPR-II is selectively transported to the basolateral surface by endogenous sorting proteins.

Sorting to the basolateral membrane surface depends on the basolateral sorting adaptor complex AP1, of which AP1µ1 is a key component that binds to cargo proteins. To examine the molecular interaction between BMPR-II and AP1µ1, we subjected cell lysates from MDCK-BR2 cells to immunoprecipitation with an anti-HA antibody. After withdrawal of Tet from culture media, AP1 µ1 coimmunoprecipitated with BMPR-II, as determined by immunoblot analysis using an anti-AP1 µ1 antibody (Fig. 2G). These findings suggest that BMPR-II associates with AP1 µ1 in polarized epithelial cells. However, this interaction was not observed in human embryonic kidney cells (HEK293) transfected with expression plasmids encoding epitope-tagged BMPR-II and AP1 µ1A (data not shown), indicating either that these proteins interact indirectly or that in HEK293 cells they lack protein modifications required for the interaction.

### Role of AP1 in Basolateral Trafficking of BMPR-II

**Figure 3:**
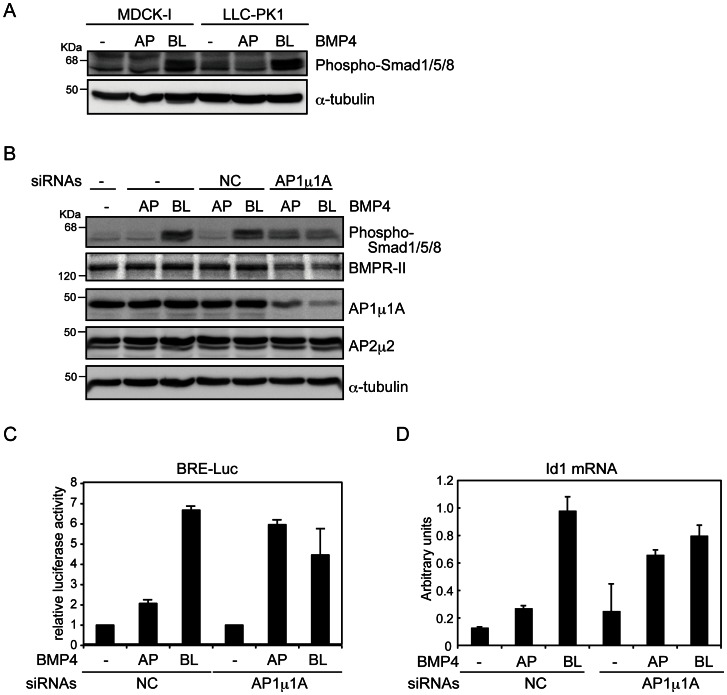
Roles of AP1 µ1A in basolateral trafficking of BMPR-II. (**A** and **B**) MDCK-I cells and LLC-PK1 cells transfected with either control (NC) or AP1 µ1A siRNAs were grown to confluence on Transwell plates, and then treated with 20 ng/ml BMP4 from the apical (AP) or basolateral (BL) sides for 45 min. Phosphorylation of Smad1/5/8 induced by BMP4 was examined by immunoblot analyses using the indicated antibodies. (**C** and **D**) LLC-PK1 cells and LLC-PK1 cells transfected with BMP-responsive element (BRE)-reporter construct in combination with either control siRNA (NC) or AP1 µ1A siRNA were seeded on Transwell plates and grown to confluence. Cells were treated with BMP4 (20 ng/ml) from the apical (AP) or basolateral (BL) sides for 2 h for luciferase assays, normalized to activity of sea pansy luciferase encoded by pRL-TK-Renilla (C), and for 1 h for quantitative RT-PCR analysis, normalized to the amounts of GAPDH mRNA (D). Each value represents the mean ± S.D. of triplicate determinations from a representative experiment. Similar results were obtained in two independent experiments (C and D).

Next, we performed knockdown experiments using siRNAs against AP1 µ1. MDCK-I cells express both AP1A and AP1B complexes, whereas LLC-PK1 cells lack the AP1B complex due to loss of the AP1 µ1B gene [19]. Like MDCK cells, LLC-PK1 cells expressed only long-form BMPR-II (Fig. 2A) and phosphorylated Smad1/5/8 only in response to basolateral treatment with BMP4 (Fig. 3A), suggesting that AP1 µ1A alone is sufficient for basolateral trafficking of BMPR-II. Endogenous AP1 µ1A was effectively knocked down by siRNAs in LLC-PK1 cells, without the affecting protein level of AP2 µ2, which is closely related to AP1 µ1 (Fig. 3B). Knockdown of AP1 µ1A altered the restricted responsiveness to BMP4; phosphorylation of Smad1/5/8 was induced by apical treatment with BMP4, but the induction by basolateral treatment was lower, probably due in part to reduced protein levels of BMPR-II in AP1 µ1A-knockdown cells (Fig. 3B). We confirmed similar effects using another AP1 µ1A siRNA (Fig. S2A). Consistent with the Smad1/5/8 phosphorylation profiles, AP1 µ1A siRNA increased the responsiveness to apical treatment with BMP4, as determined by BMP-induced activity of the luciferase reporter (BRE-Luc) and induction of endogenous Id1, a representative target gene of BMPs (Figs. 3C and 3D). In addition, sorting to the apical membrane surface of BMPR-II by AP1 µ1A siRNAs was confirmed by cell surface biotinylation analyses (Fig. S2B). Although previous studies showed basolateral localization of TGF-β receptors in MDCK cells [25], [26], we found that TGF-β induced phosphorylation of Smad2 (a TGF-β receptor–regulated Smad) in cells treated from the apical side, but not from the basolateral side, and this induction was not affected by AP1 µ1A knockdown (Fig. S2C), suggesting that cell polarity is still maintained in the AP1 µ1A-depleted cells. Knockdown of AP1 µ1A alone by specific siRNAs in MDCK-I cells, in which both AP1A and AP1B are expressed, only modestly affected selectivity of BMP4 signal input (Fig. S2D). Furthermore, MDCK-BR2 cells transfected with AP1 µ1A siRNAs showed diffuse expressions of BMPR-II-HA in the cells (Fig. S2E). These findings suggest that AP1A makes only a partial contribution to basolateral sorting of BMPR-II. Overall, these results demonstrate that basolateral sorting of BMPR-II is regulated by AP1 complexes.

### Roles of BMP in Polarized Epithelial Cells

**Figure 4:**
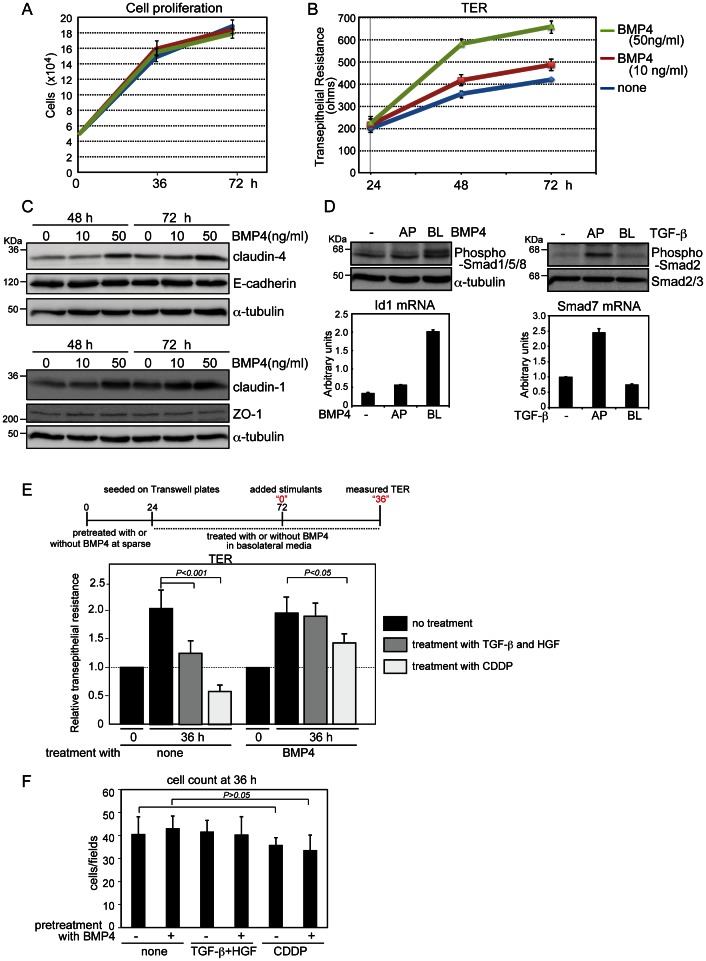
Increase of transepithelial resistance (TER) by BMP4 treatment. (**A, B,** and **C**) MDCK-I cells pretreated with BMP4 for 24 h were seeded on Transwell plates in basolateral media containing BMP4, and incubated until 72 h (i.e., for an additional 48 h). Cell counting (A), TER measurement (B), and immunoblot analyses (C) were performed at the indicated time points. (D) MDCK-I cells were grown to confluence on Transwell plates and treated for 45 min with 20 ng/ml BMP4 (left) and 1 ng/ml TGF-β (right) from the apical (AP) or basolateral (BL) sides. Phosphorylation of Smads and expression of representative target genes (Id1 for BMP4 and Smad7 for TGF-β) were examined by immunoblot and quantitative RT-PCR analyses, respectively. Each value represents the mean ± S.D. of triplicate determinations from a representative experiment. Similar results were obtained in two independent experiments (A, B and D). (**E** and **F**) MDCK-I cells pretreated with BMP4 under sparse conditions for 24 h were seeded in triplicate on Transwell plates in basolateral media containing 50 ng/ml BMP4 for 48 h, and TER was measured (indicated as “0”). Then the cells were treated for 36 h either with both 1 ng/ml TGF-β from the apical side and 10 ng/ml HGF from the basolateral side or with 25 µM CDDP from the basolateral side. After TER of all three Transwell plates had been measured at four points for each well (E), the cells from two plates were used for cell counting (F) and the other was used for E-cadherin staining (Fig. S3). Each value represents the mean±S.D. of duplicate determinations from a representative experiment. Similar results were obtained in three independent experiments.

To gain insight into the functions of BMP in polarized epithelial cells, we administered BMP4 on the basolateral side of MDCK-I cells plated on Transwell plates, and then measured TER. Cell proliferation was not significantly affected until 72 h after BMP4 treatment (Fig. 4A), although TER was slightly increased in BMP4-treated cells compared to the level in untreated control cells. A higher concentration of BMP4 (50 ng/ml) caused greater increases in TER at the time points examined (Fig. 4B), consistent with recent work reporting that Dragon, a neural adhesion protein, enhances BMP signaling and increases TER in medullary collecting duct mICD3 cells [27]. BMP4 upregulated the expression levels of proteins of the tight-junction components claudin-1 and claudin-4, both of which are indispensable for barrier function of MDCK-I cells [28], whereas the levels of E-cadherin and ZO-1 were not affected by BMP4 treatment (Fig. 4C).

As already shown in Figure 1C, BMP4 induced Smad1/5/8 phosphorylation and Id1 transcription in MDCK-I cells only when administered to the basolateral surface (Fig. 4D, left). By contrast, as in LLC-PK1 cells (Fig. S2C), TGF-β transmitted signals from the apical surface of MDCK-I cells, as determined by Smad2 phosphorylation and by the mRNA level of Smad7, a representative early target gene of TGF-β, measured using quantitative RT-PCR analysis (Fig. 4D, right). In collaboration with other growth factors or cytokines, TGF-β induces EMT in epithelial and cancer cells [29], [30]. In light of those observations, we plated MDCK-I cells at confluence on Transwell plates and treated them with both TGF-β and HGF. Although combined treatment with TGF-β and HGF for 36 h did not markedly affect E-cadherin staining, as previously reported [29], TER was reduced in cells treated with both ligands, compared with untreated control cells (Fig. 4E). In addition, cis-diamminedichloro-platinum (CDDP), an anti-tumor drug that dissociates cell–cell contact, also reduced TER to a level lower than that at time “0” in untreated control cells (Fig. 4E) [31]. When cells were pre-treated with BMP4 followed by administration of BMP4 from the basolateral side, repression of TER by both stimulants was rendered without affecting cell number or cell morphology (Figs. 4E, 4F, and S3). These findings suggest that BMP maintains epithelial polarity by transmitting signals from the basolateral membrane surface and inhibits disorganization of the epithelial sheet mediated by EMT inducers such as TGF-β and the anti-tumor drug.

We found that BMPR-II was localized exclusively at the basolateral surface of the cell membrane in polarized epithelial cells. In MDCK cells, BMP-4, BMP-6, and BMP-9, which each bind to specific type-I receptors [32], induced Smad1/5/8 phosphorylation upon basolateral administration (Fig. 1D), suggesting basolateral localization of BMPR-Is. Several BMPR-Is, ALK-1, -2, and -3, mediate Smad1/5/8 phosphorylation in response to TGF-β [33], [34]. In this study, we also observed Smad1/5/8 phosphorylation upon apical addition of TGF-β in MDCK and LLC-PK1 cells. Although the cell-surface localization of BMPR-Is has not yet been determined, it is possible that BMPR-Is are expressed at both surfaces of the cell membrane. Thus, it will be necessary to conduct further studies of BMPR-I distribution on the cell surface and on how cells knocked down for AP1 µ1A transport BMPR-II to the apical surface. Because of the low sensitivity of commercially available anti–BMPR-II antibodies, we did not detect the basolateral localization of BMPR-II in epithelial cells in kidney or in other tissues, such as salivary and mammary glands, or in injured tissues. Moreover, germ-line mutations of the BMPR-II gene are associated with primary pulmonary hypertension [35]. Some of these mutant alleles of BMPR-II, however, exhibited no functional differences from wild-type BMPR-II in reporter assays using non-polarized epithelial Mv1Lu cells [36]. It appears that these BMPR-II mutants mislocalize in endothelial cells *in vivo*, leading to a disorder of the pulmonary vascular system. To address these issues, further efforts are needed to develop high-quality BMPR-II antibodies with adequate sensitivity for immunohistochemistry.

From the results of this study, we conclude that BMP increases TER in kidney polarized epithelial cells. In addition, BMPs transmit their signals from the basolateral surface, whereas TGF-β does so from the apical surface. The vectorial nature of BMP and TGF-β signaling is likely to play crucial roles in the maintenance of polarity in polarized epithelial cells.

## Supporting Information

Figure S1 Cell morphology of MDCK-Ras cells. MDCK-Ras cells were cultured in the absence or presence of tetracycline (Tet), and analyzed by a phase-contrast microscope or confocal lase scanning microscope after staining with anti-E-cadherin or anti-ZO-1 antibodies. TOPRO was used to visualize the nucleus. Scale bars indicate 50 µm.
(TIF)

Figure S2 Roles of AP1 µ1A in basolateral trafficking of BMPR-II. (**A**) LLC-PK1 cells were transiently transfected with either control siRNA (NC) or AP1 µ1A siRNAs (#1 and #2) in 60-mm plates. After 12 h, the cells were trypsinized, seeded on Transwell plates, and grown to confluence. BMP4 (20 ng/ml) was added into apical (AP) or basolateral (BL) media and incubated for 45 min. The cells were harvested and assayed for immunoblot analyses using the indicated antibodies. (**B**) LLC-PK1 cells transfected with either control siRNA (NC) or AP1 µ1A siRNAs (#1 and #2) were seeded on Transwell plates, and biotinylated from the apical (AP) or basolateral (BL) sides. Equal quantities of proteins were subjected to SDS-PAGE (total cell lysate), or incubated with Streptavidin Sepharose 4B to isolate biotinylated proteins, followed by SDS-PAGE. (**C**) TGF-β (1 ng/ml) was added into apical (AP) or basolateral (BL) media and incubated for 45 min. The cells were harvested and subjected to immunoblot analyses using the indicated antibodies. (**D**) MDCK-I cells were transiently transfected with either control siRNA (NC) or AP1 µ1A siRNAs (#d1 and #d2) in 6-well plates. After 12 h, the cells were trypsinized and seeded on Transwell plates and grown to confluence. BMP4 (20 ng/ml) was added into apical (AP) or basolateral (BL) media and incubated for 45 min. Cells were harvested and assayed for immunoblot analyses using the indicated antibodies. (**E**) MDCK-BR2 cells were transfected with either control siRNA (NC) or AP1 µ1A siRNAs (#d1 and #d2) and examined by immunoblot analyses and Matrigel culture in the presence or absence of tetracycline (Tet), followed by staining with an anti-HA antibody (green), rhodamine-phalloidin (red), and TOPRO (white).
(TIF)

Figure S3 Effects of TGF-β/HGF and CDDP treatments on MDCK-I cells. MDCK-I cells pretreated with BMP4 under sparse conditions for 24 h were seeded in triplicate on Transwell plates in the basolateral media containing 50 ng/ml BMP4 for 48 h. The cells were treated with both 1.0 ng/ml TGF-β from the apical side and 10 ng/ml HGF from the basolateral side, or with 25 µM CDDP from basolateral side for 36 h. After TER was measured, the cells from two Transwell plates were used for cell counting (Fig. 4F), and cells from the other Transwell were used for E-cadherin staining. TOPRO was used to visualize the nucleus.
(TIF)

Table S1 The primers used in the present study are shown.
(TIFF)
