# Supplementary figures and images for: Basolateral BMP Signaling in Polarized Epithelial Cells
Source: PLoS One. 2013 May 13;8(5):e62659. doi: 10.1371/journal.pone.0062659 (PMC3652834; doi:10.1371/journal.pone.0062659)

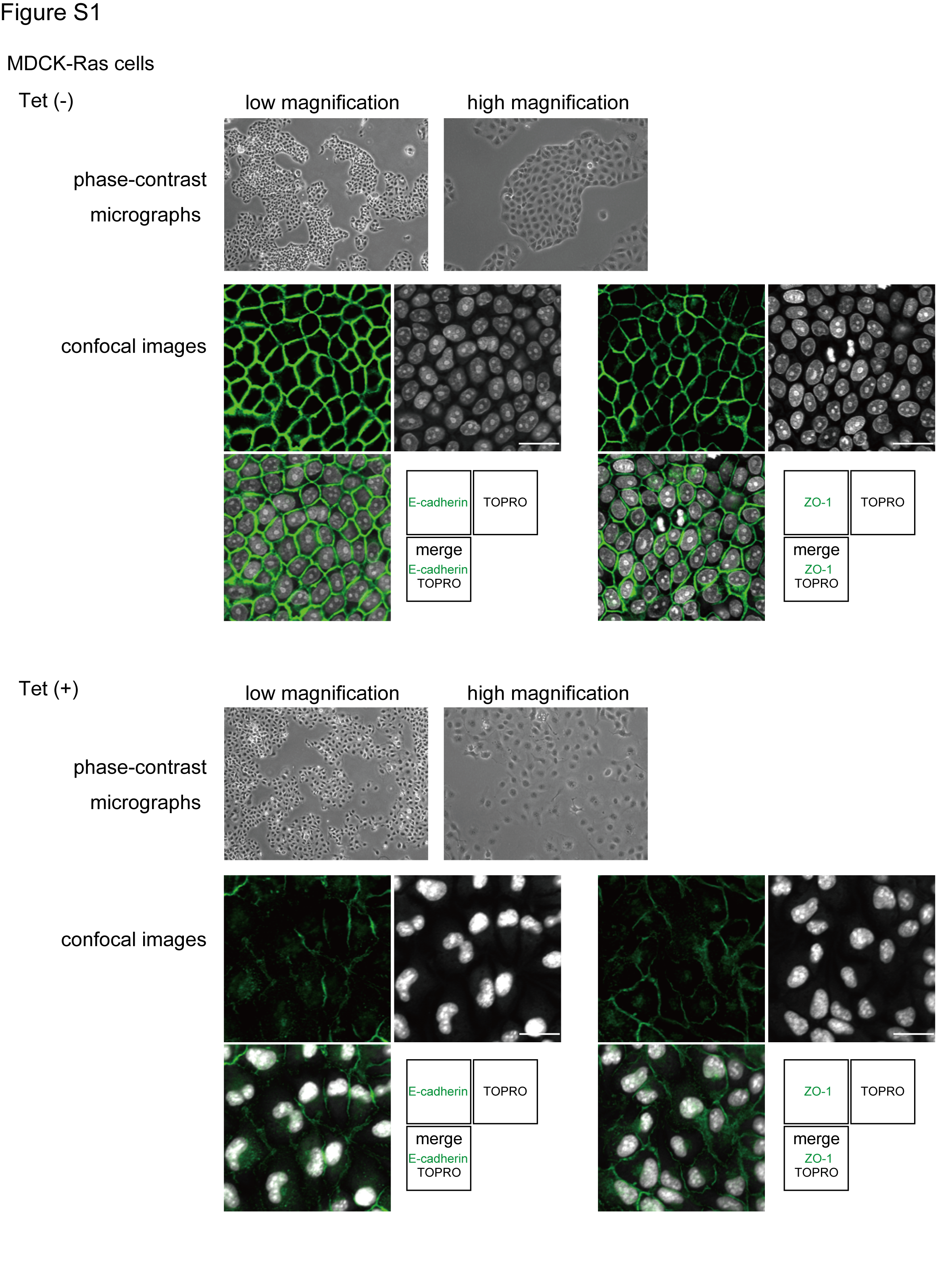

Supplement: Figure S1 — Cell morphology of MDCK-Ras cells. MDCK-Ras cells were cultured in the absence or presence of tetracycline (Tet), and analyzed by a phase-contrast microscope or confocal lase scanning microscope after staining with anti-E-cadherin or anti-ZO-1 antibodies. TOPRO was used to visualize the nucleus. Scale bars indicate 50 µm. (TIF) [file pone.0062659.s001.tif]

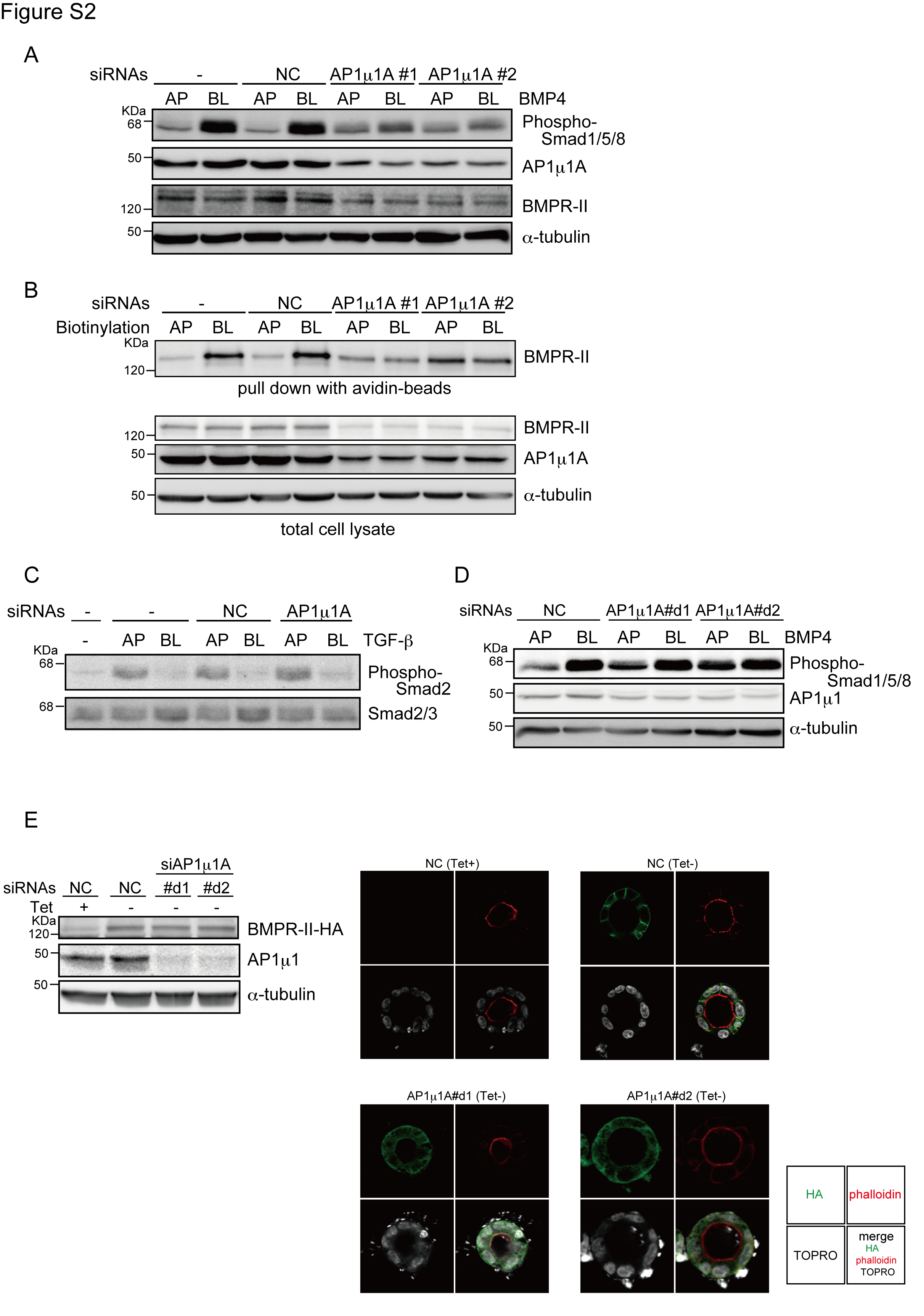

Supplement: Figure S2 — Roles of AP1 µ1A in basolateral trafficking of BMPR-II. (A) LLC-PK1 cells were transiently transfected with either control siRNA (NC) or AP1 µ1A siRNAs (#1 and #2) in 60-mm plates. After 12 h, the cells were trypsinized, seeded on Transwell plates, and grown to confluence. BMP4 (20 ng/ml) was added into apical (AP) or basolateral (BL) media and incubated for 45 min. The cells were harvested and assayed for immunoblot analyses using the indicated antibodies. (B) LLC-PK1 cells transfected with either control siRNA (NC) or AP1 µ1A siRNAs (#1 and #2) were seeded on Transwell plates, and biotinylated from the apical (AP) or basolateral (BL) sides. Equal quantities of proteins were subjected to SDS-PAGE (total cell lysate), or incubated with Streptavidin Sepharose 4B to isolate biotinylated proteins, followed by SDS-PAGE. (C) TGF-β (1 ng/ml) was added into apical (AP) or basolateral (BL) media and incubated for 45 min. The cells were harvested and subjected to immunoblot analyses using the indicated antibodies. (D) MDCK-I cells were transiently transfected with either control siRNA (NC) or AP1 µ1A siRNAs (#d1 and #d2) in 6-well plates. After 12 h, the cells were trypsinized and seeded on Transwell plates and grown to confluence. BMP4 (20 ng/ml) was added into apical (AP) or basolateral (BL) media and incubated for 45 min. Cells were harvested and assayed for immunoblot analyses using the indicated antibodies. (E) MDCK-BR2 cells were transfected with either control siRNA (NC) or AP1 µ1A siRNAs (#d1 and #d2) and examined by immunoblot analyses and Matrigel culture in the presence or absence of tetracycline (Tet), followed by staining with an anti-HA antibody (green), rhodamine-phalloidin (red), and TOPRO (white). (TIF) [file pone.0062659.s002.tif]

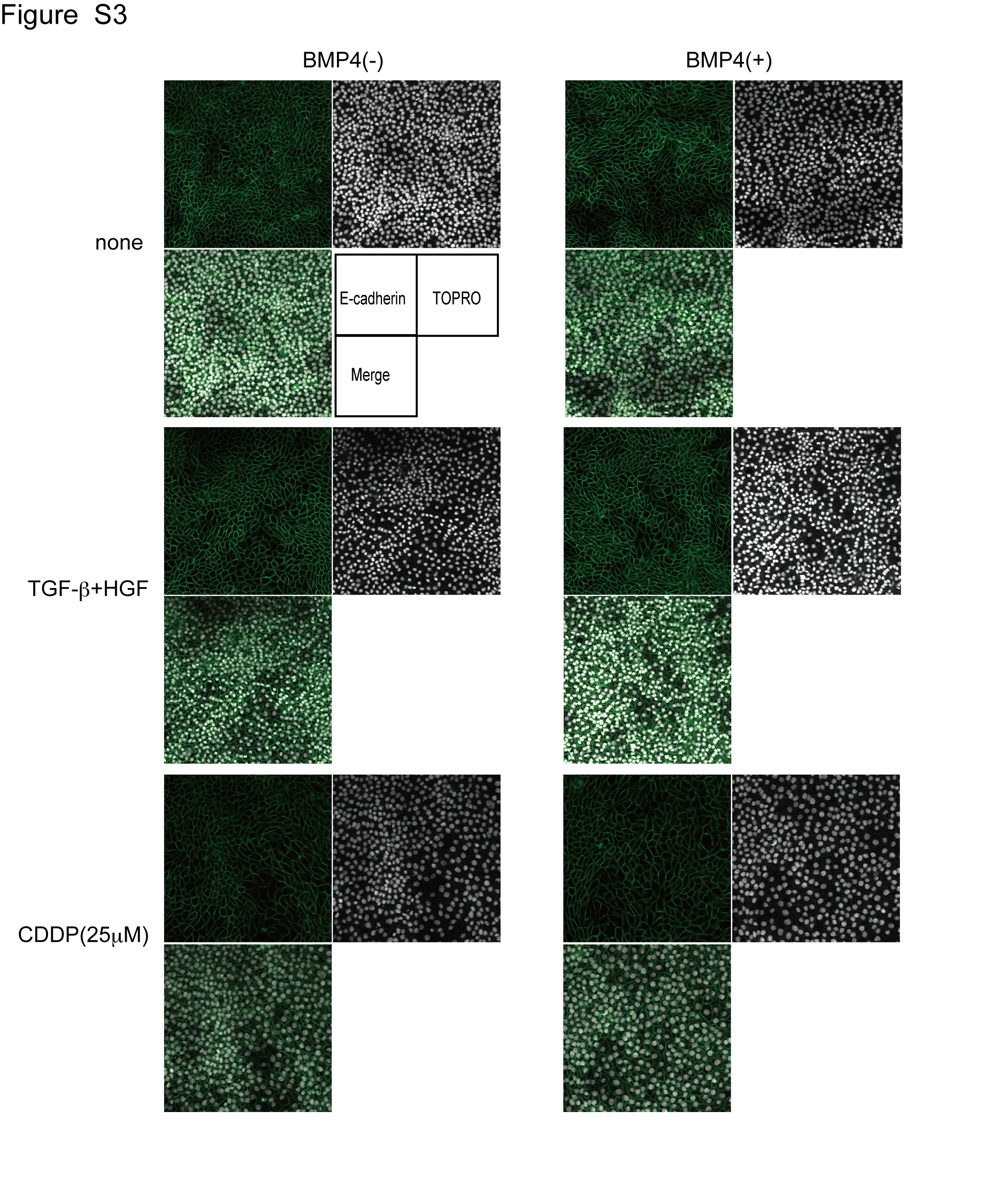

Supplement: Figure S3 — Effects of TGF-β/HGF and CDDP treatments on MDCK-I cells. MDCK-I cells pretreated with BMP4 under sparse conditions for 24 h were seeded in triplicate on Transwell plates in the basolateral media containing 50 ng/ml BMP4 for 48 h. The cells were treated with both 1.0 ng/ml TGF-β from the apical side and 10 ng/ml HGF from the basolateral side, or with 25 µM CDDP from basolateral side for 36 h. After TER was measured, the cells from two Transwell plates were used for cell counting (Fig. 4F), and cells from the other Transwell were used for E-cadherin staining. TOPRO was used to visualize the nucleus. (TIF) [file pone.0062659.s003.tif]
